# Deaths in Philadelphia from June 22d to July 20th, 1850

**Published:** 1850-08

**Authors:** James Aitken Meigs

**Affiliations:** Student of Medicine


*Deaths in Philadelphia from June 22d to July 20th,* 1850. Reported by Mr. James Aitken Meigs, Student of Medicine.

| Diseases. | Ad'ts | Chil. | Diseases. | Ad'ts | Chil. |
|---|---|---|---|---|---|
| Anæmia, . . . | 0 | 1 | Fever, typhoid . . | 3 | 3 |
| Aphthæ, . . . . | 0 | 3 | " typhus, . . | 3 | 1 |
| Apoplexy, . . . | 14 | 0 | Fracture of skull, . . | 0 | 2 |
| Asphyxia, . . . | 0 | 2 | Gangrene of foot, . . | 1 | 0 |
| Asthma, . . . . | 0 | 1 | " lungs . . | 1 | 1 |
| Burns and Scalds, . . | 1 | 3 | Hæmoptysis, . . . | 1 | 0 |
| Cachexia, . . . | 0 | 1 | Hemorrhage from gums, . | 0 | 1 |
| Cancer, . . . . | 1 | 0 | Hernia, . . . . | 3 | 0 |
| " breast, . . | 1 | 0 | Hydrocephalus, . . | 0 | 36 |
| " liver, . . | 1 | 0 | Hydropericardium, . . | 1 | 0 |
| " stomach, . | 3 | 0 | Hydrothorax, . . . | 1 | 1 |
| " throat, . . | 1 | 0 | Inanition, . . . . | 0 | 7 |
| Caries of spine, . . | 0 | 1 | Inflammation of brain, . | 6 | 25 |
| Casualties, . . . | 7 | 9 | " bronchi, . . | 4 | 6 |
| Cholera, . . . . | 4 | 0 | " heart, . . | 1 | 1 |
| Cholera infantum, . . | 0 | 211 | " kidneys, . . | 1 | 1 |
| " morbus, . . | 7 | 5 | " larynx, . . | 0 | 3 |
| Concussion of brain, . | 0 | 1 | " liver, . . | 2 | 1 |
| Congestion of lungs, . | 0 | 4 | " lungs, . . | 5 | 15 |
| " brain, . | 3 | 13 | " peritoneum, . | 1 | 1 |
| Convulsions, . . . | 1 | 60 | " stom. & bowels, | 5 | 14 |
| " puerperal, . | 2 | 0 | " uterus, . . | 2 | 0 |
| Croup, . . . . | 0 | 4 | Intemperance, . . | 3 | 0 |
| Cyanosis, . . . | 0 | 3 | Intussusception, . . | 1 | 0 |
| Debility, . . . | 3 | 11 | Jaundice, . . . | 0 | 2 |
| Dementia, . . . | 1 | 0 | Malformation, . . | 0 | 4 |
| Diabetes, . . . | 2 | 0 | " of heart, . | 0 | 1 |
| Diarrhœa, . . . | 13 | 30 | Mania-a-potu, . . | 9 | 0 |
| Disease of brain, . . | 2 | 11 | Marasmus, . . . | 1 | 42 |
| " chest, . . | 0 | 1 | Measles, . . . | 0 | 4 |
| " heart, . . | 7 | 1 | Old age, . . . . | 19 | 0 |
| " liver, . . | 0 | 1 | Palsy, . . . . | 4 | 0 |
| " lungs, . . | 0 | 4 | Perforation of intestines, . | 0 | 1 |
| " spine, . . | 0 | 1 | Phthisis pulmonalis, . | 44 | 16 |
| " stomach and bowels, | 0 | 6 | Pertussis, . . . | 0 | 13 |
| Dropsy, . . . | 5 | 3 | Poisoning, . . . | 0 | 1 |
| " abdominal, . | 1 | 0 | Scirrhus of liver, . . | 1 | 0 |
| Drowned, . . . | 7 | 9 | Scrofula, . . . | 0 | 3 |
| Dysentery, . . . | 22 | 43 | Small pox, . . . | 3 | 6 |
| Effusion on brain, . . | 1 | 9 | Still born, . . . | 0 | 47 |
| Enlargement of heart, . | 1 | 0 | Spina bifida, . . . | 0 | 1 |
| Epilepsy, . . . | 1 | 0 | Spasm of intestines, . | 0 | 1 |
| Erysipelas, . . . | 0 | 3 | Suicide, . . . . | 2 | 0 |
| Exhaustion, . . . | 0 | 1 | Sunstroke, . . . | 0 | 1 |
| Fever, . . . . | 2 | 2 | Tabes mesenterica, . | 0 | 2 |
| " intermittent, . | 0 | 1 | Teething, . . . | 0 | 2 |
| " puerperal, . . | 2 | 0 | Tetanus, . . . | 1 | 0 |
| " remittent, . . | 3 | 2 | Tuberculosis, . . . | 0 | 4 |
| " scarlet, . . | 2 | 32 | Tumor of larynx, . . | 0 | 1 |

| Diseases. | Ad'ts | Chil. | Diseases. | Ad'ts | Chil. |
|---|---|---|---|---|---|
| Ulceration of throat . | 0 | 1 | Violence, . . . | 1 | 0 |
| " bowels, . | 2 | 0 | Wounds, . . . | 0 | 1 |
| Unknown, . . . | 5 | 9 | | | |
| | | | | 258 | 776 |

Total, . . . . . . . . . . . 1034

Of the foregoing the ages were as follows:—

| | | | | | |
|---|---|---|---|---|---|
| Under | 1 year, | - | | - - | 470 |
| From | 1 | to - | 2, | - - | 152 |
| | 2 | - - | 5, | - - | 67 |
| | 5 | - - | 10, | - - | 45 |
| | 10 | - - | 15, | - - | 18 |
| | 15 | - - | 20, | - - | 24 |
| | 20 | - - | 30, | - - | 66 |
| | 30 | - - | 40, | - - | 55 |
| | 40 | - - | 50, | - - | 38 |
| | 50 | - - | 60, | - - | 38 |
| | 60 | - - | 70, | - - | 28 |
| | 70 | - - | 80, | - - | 25 |
| | 80 | - - | 90, | - - | 6 |
| | 90 | - - | 100, | - - | 2 |
| | | | | | 1034 |

Included in this number, are 66 from the Almshouse, 16 from the surrounding country, and 18 people of color.

